# FTIR-Microspectroscopy Detection of Metronidazole Teratogenic Effects on Mice Fetus 

**Published:** 2014

**Authors:** Azadeh Ashtarinezhad, Farshad H. Shirazi, Hossein Vatanpour, Baharak Mohamazadehasl, Ataolla Panahyab, Maryam Nakhjavani

**Affiliations:** a *Department of Toxicology, School of Pharmacy, Shahid Beheshti University of Medical Sciences, Tehran, Iran.*; b *Pharmaceutical Sciences Research Center, Shahid Beheshti University of Medical Sciences, Tehran, Iran.*; c *Young Researchers Club, Science and Research Branch, Islamic Azad University, Tehran, Iran. *

**Keywords:** FTIR-micro spectroscopy, Metronidazole, Teratogenic, Mice Fetus, Bio-spectroscopy

## Abstract

Metronidazole is used to treat trichomoniasis, bacterial vaginosis, and other diseases. There are controversy aspects about its teratogenicity. A teratogenic agent can alter morphology or subsequent function of the fetus. The aim of this study was to examine an alternative method for the recognition of the mechanism or the bimolecular potential changes in mice fetus caused by Metronidazole using FTIR micro spectroscopy. The mice were injected with metronidazole (60 mg/Kg) on gestation day 9. Fetuses were dissected on day 15 of gestation and morphological and histological studies on the fetus were carried out. Serial sectioning (10 μm) of normal and metronidazole-treated brains and livers were used for FTIR measurement in the wave number region of 600- 3600 cm^-1^.The results showed that there were some variations between the fetus of normal and treated brain and liver. The band intensities in fetus brain and liver of test animals were reduced and shifted at 707 cm^-1^, 1155 cm^-1^, 1054 cm^-1^, 1256 cm^-1^ and 1219 cm^-1^, 1453 cm^-1^ and 1525 cm^-1^, 1622 cm^-1^, 1645 cm^-1^ and 2944 cm^-1^,while the band intensities were increased and shifted at 879 cm^-^^1^ , 810 cm^-1^, 1223 cm^-1^, 1256 cm^-1^ 1360 cm^-1^, 1723 cm^-1^. It was concluded that most of variations in brain and liver of Metronidazole treated fetuses are in amid bands, nucleic acid and carbohydrate related bands. Based on these findings FTIR spectroscopy can be a useful tool for bio diagnostic.

## Introduction

Teratogenecity is to study the fetus defects caused by a treatogenic agent. A teratogenic agent is a chemical, infectious agent, physical condition, or deficiency that when exposed to fetus can alter the morphology or subsequent biological function of the delivered baby (1). The embryo is more susceptible to the teratogenic agents during the period of rapid differentiation. The type of congenital malformation produced by an exposure depends upon which organ is most susceptible at the time of the exposure to the teratogenic agent (2). Teratogenicity studies and investigations, however, are very much limited to the obvious malformations apparent during a short period of life after birth. Many of late expressing biological changes caused by teratogens are ignored basically due to the lack of proper diagnostic instruments to be able to trigger the non-obvious changes in organs’ biomolecules during the embryonic life.

Metronidazole as a derivative of nitroimidazole is active against anaerobic bacteria and protozoa (3). This medication contains a nitro-group that causes its toxicity on microbial cells (4). Metronidazole is mostly used to treat trichomoniasis, bacterial vaginitis, and other similar diseases. As is the case with many drugs, physicians are not sure on its teratogenicity, but often hesitate to use it during pregnancy, particularly in the first trimester (5). Teratogenic effects of Metronidazole are rather controversial (6). While controlled studies of Metronidazole in rats, mice, and rabbits did not show teratogenic effects (7, 8), there are evidences on the embryotoxic and teratogenic effects in rats, mice, and guinea pigs at dose levels corresponding to the human dose (9-11). Animal studies with oral Metronidazole showed increased incidences of tumor in the lung, liver, testes, reticulum, mammary gland and pituitary gland in certain rodent species (12-14). In spite of earlier studies which suggested a relation between Metronidazole and various birth defects, more recent studies do not support a significant increased risk for birth defects on the fetus (15, 16). Skeletal malformations reported by Metronidazole are such as delayed development and out of embryo heart formation which is observed by different imidazole and triazole derivatives (17-19).

The vibrational spectroscopic techniques, including FTIR micro spectroscopy, are potential tools for noninvasive optical tissue diagnosis. In recent years, FTIR spectroscopy has proven itself as a potential technique in presenting the chemical features of living biological samples (20-22). The present status of this technique is to examine this method in different areas and to measure its applicability for various pathologic conditions. Firmed convincing results have been published on the use of this technique to distinguish between normal and abnormal conditions in malignancy (23-24), and discriminative results for other tissues are coming out gradually (25). It is important to consider the preliminary steps of this approach, so that a rough idea is expected for any conditions with specific results coming later on.

Due to the controversial nature of the teratogenic effects reported for different agents, and to the extensive time consuming and costly methodologies of teratogenic investigations, as well as the missing of many non-obvious biomolecular alterations in fetus with the present facilities, we have started a series of investigations to evaluate the applicability of FTIR spectroscopy for the determination of different agents. The aim of this study was to examine an alternative method for the detection of any changes in mice 15 days fetus which mother has been exposed to Metronidasole compare to the control animals at the spectral regions related to organs lipid, protein, and DNA biomolecules using FTIR micro spectroscopy.

## Experimental

*Tissue preparation and morphology*

Adult mice (10-12 weeks) weighting 20 g were obtained from Razi institute, Iran. The mice were fed with a standard diet with water and libitum, and kept in a room with controlled light (12:12, light: dark), temperature (22 °C), relative humidity (45%) and ventilation (15 air changes/ hour). They were allowed to adapt to their environment for 1 week prior to the experiments. The mice were randomly mated and for emphasis of pregnancy were checked vaginal plaque after their mating. Then, they were randomly distributed in 2 groups: group 1- control and group 2- Metronidazole (which received 60 mg/Kg/day drug IP on gestation day 9 of pregnancy) treated. Pregnant mice were sacrificed and dissected on day 15th of gestation and morphological and histological studies on the embryos were carried out. Measurements of embryos weight were accomplished by digital balance. Crown-Rump (C-R) lengths were accomplished by coils. Serial sections were performed at a pre-defined thickness of 10 μm after fetus fixation with a fixative solution. Slices were either thaw-mounted on a 1 mm thick KBr window for IR micro spectroscopy or were mounted on conventional glass slides for staining with haematoxylin and eosin (H and E) for studying of abnormalities in embryo by light microscopy. H and E staining is the most widely used to stain in medical diagnosis which colors nuclei of cells blue and cellular eosinophilic structures in various shades of red, pink and orange (26). 

*FTIR micro spectroscopy*

FTIR measurements were performed in the absorbance mode. WQF-510 Fourier transform spectrometer (Rayleigh Optics, China) equipped with a KBr beam splitter and DLa TGS (deuterated Lanthanide triglycine sulphate) detector and μMAX IR microscope (PIKE Technologies, USA). The spectra were scanned in the mid-IR range from 400 to 4000 cm^-1^, with a resolution of 4 cm^-1^. 100 scans were recorded for each spectrum and the spectra were corrected against the background spectrum. 

*Data processing and analysis:*

The data were analyzed using routines of the Main FTOS IR software. The spectra were normalized after the baseline correction of the entire spectrum. The spectra were recorded from several sites on the liver and brain tissues of mice fetus sections and an average spectrum from the all spectra was computed. Second order derivatives were also calculated. Calculation of the second derivatives enhanced spectral features and also compensates for baseline shifts. From normalized absorbance spectra various spectral parameters were calculated and plotted against the x and y pixel coordinates. 

*Statistics*

The data from embryo weight and C-R length were analyzed by using SPSS statistical software (p<0.001). Also, the comparison between averages were done by standard error and One-way variances (p < 0.001). 

## Results and Discussion

*Morphologic studies*

**Figure 1:**
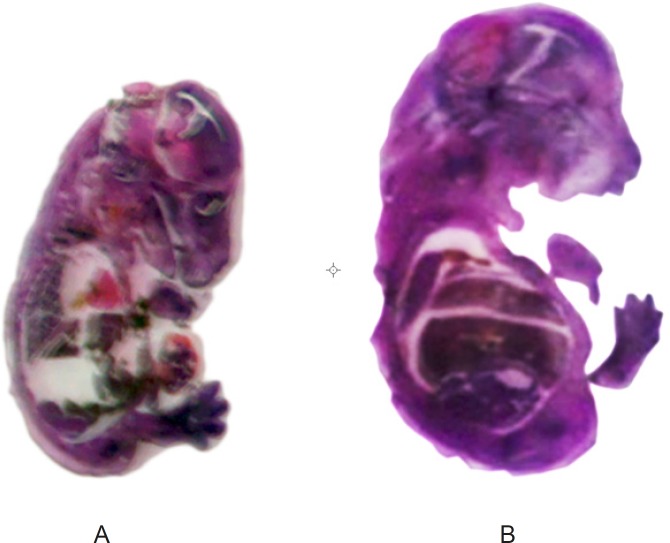
Photomicrograph of H and E- stained normal (A), metronidazole (B) mice fetus sections**. **The liver and brain were probed

**Figure 2:**
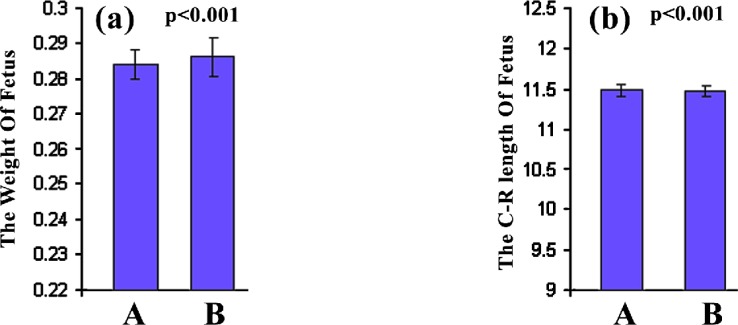
The weight of fetus (a) and the C-R length of fetus (b) in normal (A) or Metronidazole treated (B) mice as described in the methodology section

Figure 1 shows the H and E section of a Metronidazole treated mice fetuses. Clearly, the size of Metronidazole-treated fetus and specially its liver is larger than the normal fetus. In this study, the weight of treated fetus was more than the normal fetus but there wasn᾽t significant difference between them (p < 0.001) (Figure 2 (a)). The C-R length of treated-fetus was more than the normal fetus but there wasn᾽t any significant difference between them (p<0.001) (Figure 2 (b)). 

*FTIR studies to determine biomolecular changes of the liver of mouse fetus treated with metronidazole*

**Figure 3:**
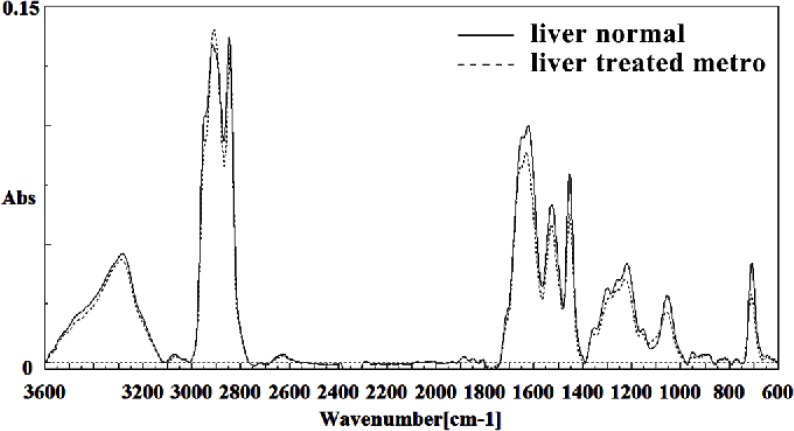
Mid-infrared spectra of normal (solid line) and Metronidazole treated (dot line) liver sections in the 600–3600 cm^–1^ wave number region

Figure 3 illustrates the typical IR spectra of the liver tissue of normal fetus exposed with Metronidazole and those exposed to Metronidazole. The information resulted in this IR absorption spectrum originates from many different types of biomolecules in the tissue, including proteins, lipids, carbohydrates, and nucleic acids. Figure 3 shows that the spectral patterns in the exposed tissue are different from those in the corresponding unexposed tissue. The most significant changes occurred in the absorbance regions from 600-3600 cm^-1^. 

**Figure 4:**
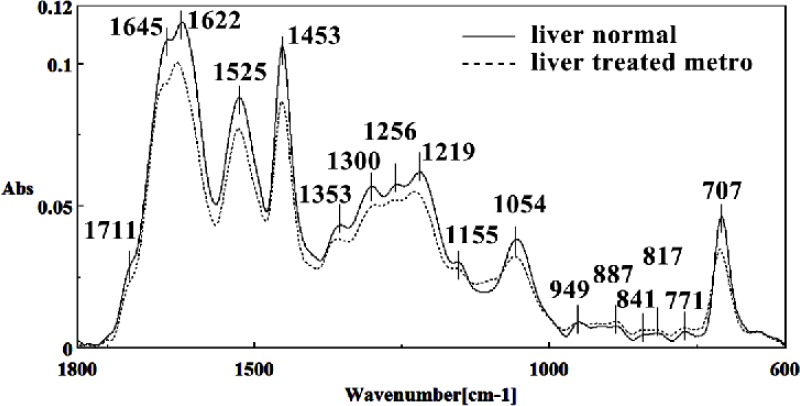
Mid-infrared spectra of normal (solid line) and Metronidazole treated (dot line) liver sections in the 600–1800 cm^–1^ wave number region

**Figure 5:**
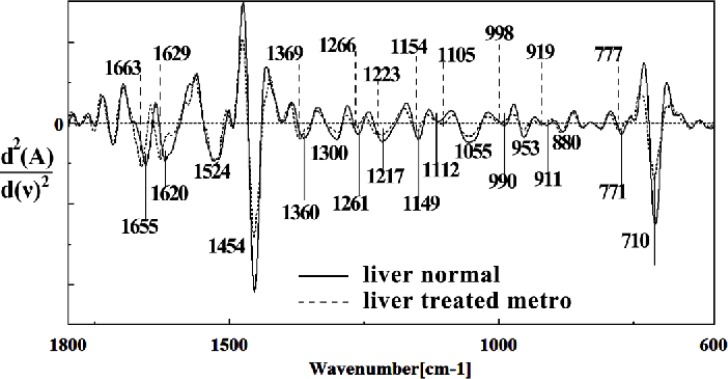
Second derivative of mean FTIR spectra of normal (solid line) and Metronidazole-treated (dot line) liver sections in the 600^–1^800 cm^–1^ wave number region

The intensity and frequency of the amide I bands around 1645 cm^-1^ in treated tissue was reduced and shifted compared to untreated tissue, mainly owing to the Amide I, C5 methylated cytosine, C=O, stretching C=C uracil, NH2. A band in treated tissue at 1622 cm^-1^ was reduced and shifted owing to Peak of nucleic acids due to the base carbonyl stretching and ring breathing mode. Moreover, the intensity and frequency of the amide II bands around 1453 cm^-1^ and 1525 cm^-1^ in treated tissues were reduced and shifted compared to untreated tissue, mainly owing to asymmetric methyl deformation and stretching C=N, C=C groups, respectively. The intensity and frequency of the bands around 1256 cm^-1^ and 1219 cm^-1^ in treated tissue were reduced and shifted compared to untreated tissue, mainly owing to PO2- asymmetric (phosphate I; 1256 cm^-1^) and PO2- asymmetric vibrations of nucleic acids when it is highly hydrogen-bonded asymmetric hydrogen-bonded phosphate stretching mode (1219 cm^-1^) (Figure 4 and 5). 

**Figure 6:**
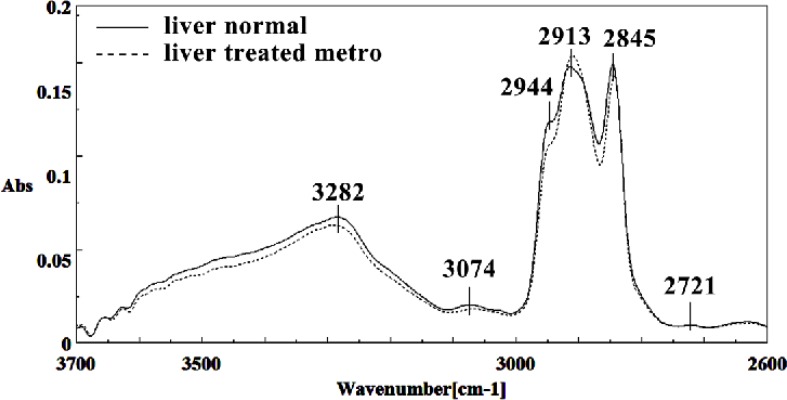
Mid-infrared spectra of normal (solid line) and Metronidazole treated (dot line) mice fetus liver sections in the 2600–3700 cm^–1^ wave number region

**Figure 7:**
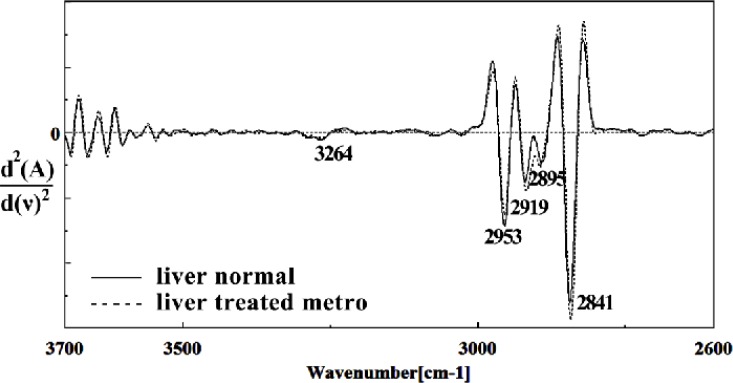
Second derivative of mean FTIR spectra of normal (solid line) and Metronidazole-treated (dot line) liver sections of mice fetus in the 2600–3700 cm^–1^ wave number region

**Figure 8:**
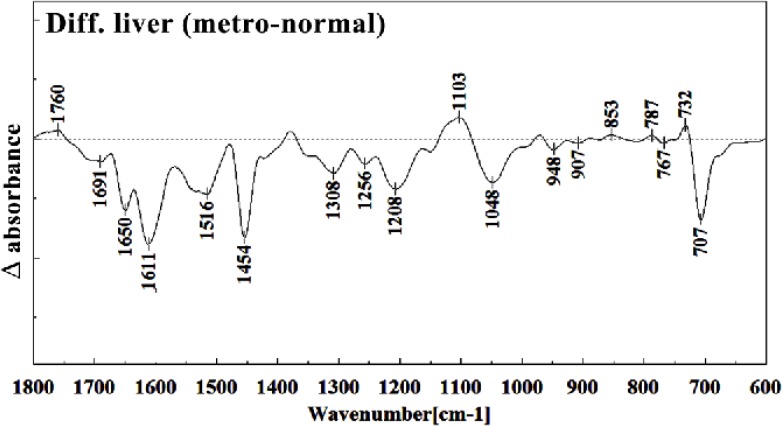
Difference FTIR spectra of Metronidazole-treated liver sections in the 600–1800 cm^–1^ wave number region from normal liver sections (Metronidazole-treated liver sections spectra-normal liver sections spectra).

**Figure 9:**
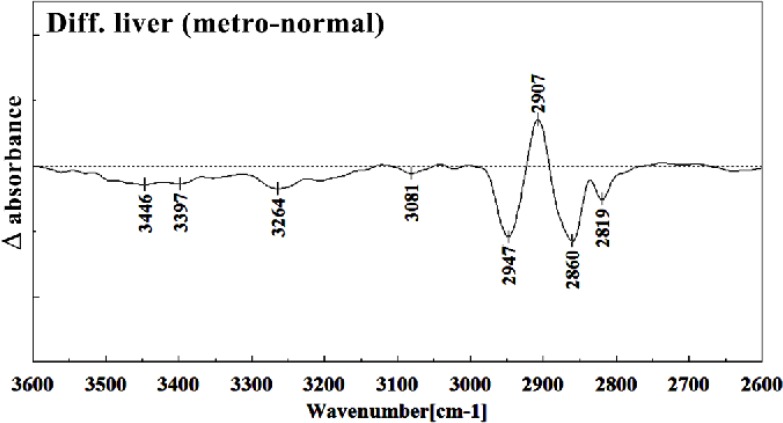
Difference FTIR spectra of Metronidazole-treated liver sections in the 2600–3700 cm^–1^ wave number region from normal liver sections (Metronidazole-treated liver sections spectra-normal liver sections spectra).

The intensities of the absorption bands near 2944 cm^-1^ (stretching C-H) in treated tissue were reduced and shifted compared to the untreated tissue (Figure 6 and 7). The intensity of the absorption band around 1054 cm^-1^ was gradually reduced and shifted due to phospholipid phosphate and partly from oligosaccharide C-OH bonds phosphate ester in Metronidazole treated tissue. The absorption band around 771 cm^-1^ in treated tissue resulted from Guanine in a C3´endo/ syn conformation in the Z conformation of DNA was shifted in treated fetus. The absorption band at 707 cm^-1^ was shifted and reduced in treated fetus owing to the Out-of-plane bending vibrations. A band at 1155 cm^-1^ in treated tissue, corresponding to the C-O stretching vibration, was also reduced and shifted (Figure 4 and 5). Absorption at bands 732 cm^-1^ (Out-ofplane bending vibrations), 853 cm^-1^ (C3׳endo/anti (A-form helix) conformation), 1103 cm^-1^ (Symmetric stretching P-O-C) and 1760 cm^-1^ were increased while the absorption at bands 907 cm^-1^ (Phosphodiester region), 948 cm^-1^ (Carotenoid), 1308 cm^-1^ (Amide III) and 1691 cm^-1^ (Peak of nucleic acids due to the base carbonyl stretching and ring breathing mode) were reduced (Figure 8). Absorption at bands 2819 cm^-1^ (Stretching N-H (NH^3^^+^), 2860 cm^-1^ (Stretching C-H), 3081 cm^-1^ (C-H ring), 3264 cm^-1^ (Stretching O-H symmetric), 3397 cm^-1^ and 3446 cm^-1^ (Stretching O-H asymmetric) were reduced in Metronidazole treated mouse fetus liv er tissue while the absorption band at 2907 cm^-1^ (Stretching vibrations of CH2 and CH3 of phospholipids, cholesterol and creatine) was increased (Figure 9).

*FTIR studies to determine biomolecular changes of the brain of mouse fetus treated with*

*metronidazole*

**Figure 10:**
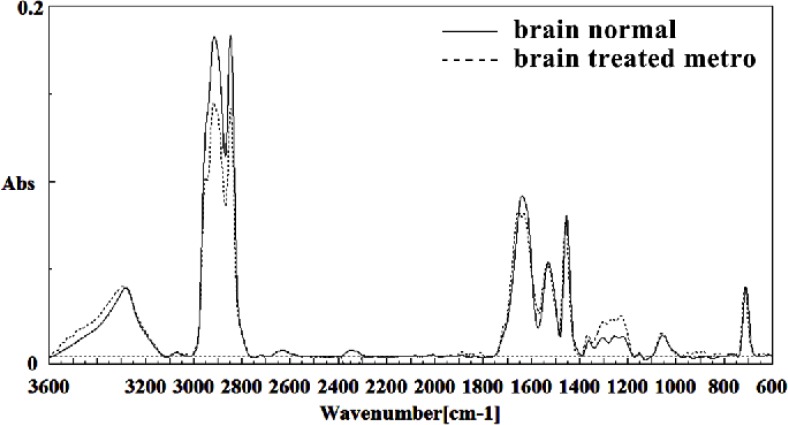
Mid-infrared spectra of normal (solid line) and Metronidazole treated (dot line) brain sections in the 600–3600 cm^–1^ wave number region

Figure 10 shows the typical IR spectra of the control group mouse fetus brain tissue compared to the brain tissue of the Metronidazole treated mouse fetus in the region of 600-3600 cm^-1^. The intensity and frequency of the amide I band at 1636 cm^-1^ in treated tissue was reduced and shifted in Metronidazole treated samples compared to untreated tissue, mainly owing to the β-sheet structure of amide I. 

**Figure 11:**
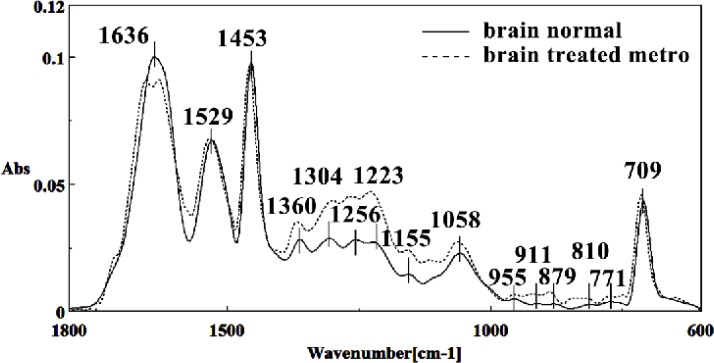
Mid-infrared spectra of normal (solid line) and Metronidazole treated (dot line) brain sections in the 600–1800 cm^–1^ wave number region

**Figure 12:**
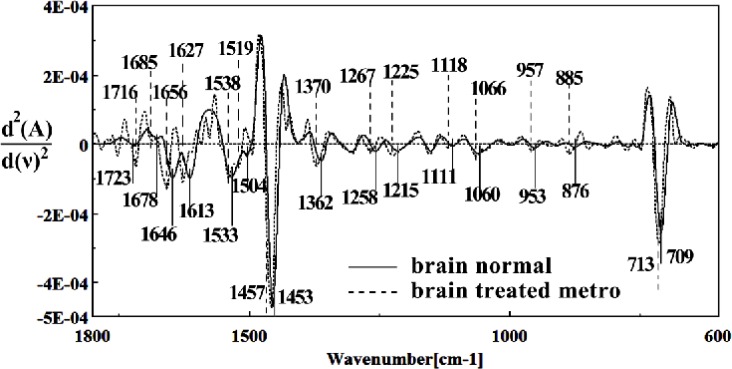
Second derivative of mean FTIR spectra of normal (solid line) and Metronidazole-treated (dot line) brain sections in the 600–1800 cm^–1 ^wave number region

This band was also broken down to two bands in treated tissue. Moreover, the intensity and frequency of the amide II bands around 1453 cm^-1^ and 1529 cm^-1^ in treated tissue were shifted compared to untreated tissue, mainly owing to asymmetric methyl deformation and C=N of adenine, and cytosine, respectively. The intensity and frequency of the bands at 1360 cm^-1^, 1256 cm^-1^ and 1223 cm^-1^ in treated tissue were increased and shifted compared to untreated tissue, mainly owing to the stretching C-O, deformation C-H,

deformation N-H (1360 cm^-1^), PO2 – asymmetric (phosphate I; 1256 cm^-1^) and PO2 – asymmetric (phosphate I; 1223 cm^-1^) groups (Figures 11 and 12). The intensity of the absorption band around 1723 cm^-1^ was gradually increased and shifted due to C=O stretching band mode of the fatty acid esters in the treated tissue. On the other hand, the intensity of C3´endo/anti (A-form helix) conformation and ring CH deformation were increased and shifted at 879 cm^-1^ and 810 cm^-1^, respectively. The absorption band around 709 cm-1was shifted and a little bit reduced in treated fetus owing to the Out-of-plane bending

vibrations.

**Figure 13:**
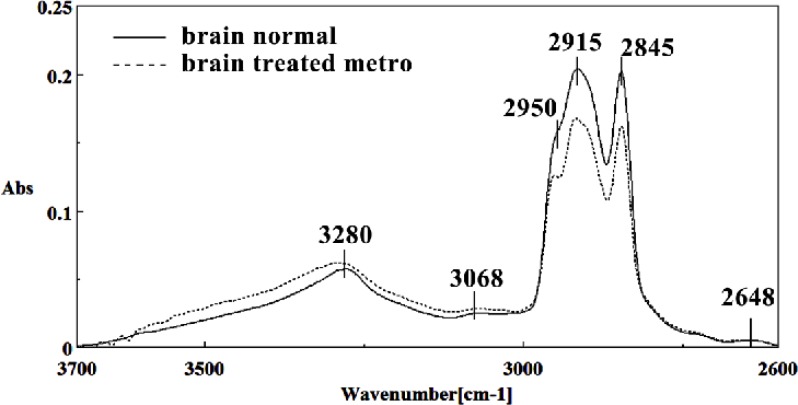
Mid-infrared spectra of normal (solid line) and Metronidazole treated (dot line) brain sections in the 2600–3700 cm^–1^ wave number region

**Figure 14:**
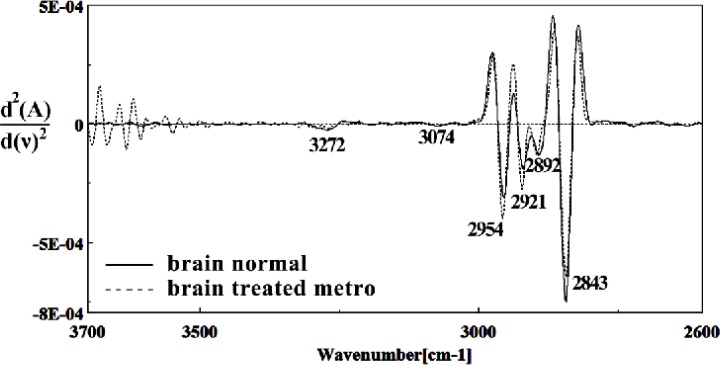
Second derivative of mean FTIR spectra of normal (solid line) and Metronidazole-treated (dot line) brain sections in the 2600–3700 cm^–1^ wave number region

**Figure 15:**
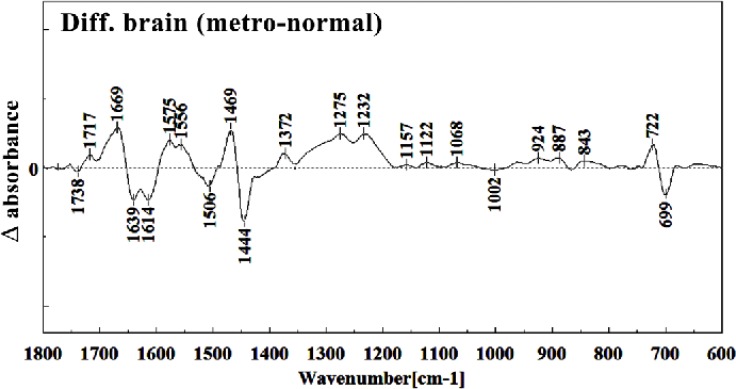
Difference FTIR spectra of Metronidazole-treated brain sections in the 600–1800 cm^–1^ wave number region from normal brain sections (Metronidazole-treated brain sections spectra-normal brain sections spectra).

**Figure 16:**
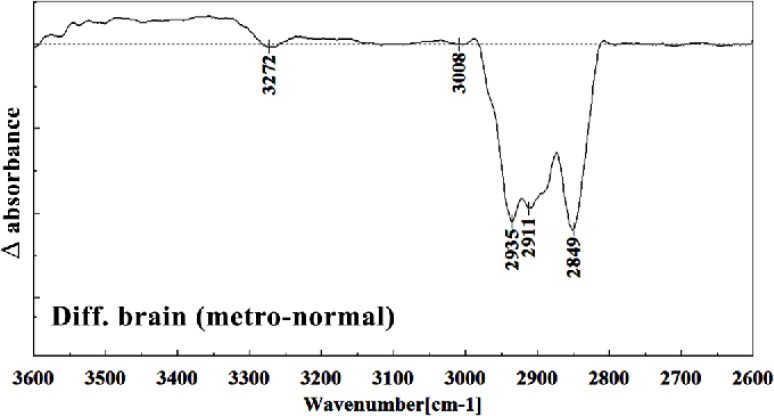
Difference FTIR spectra of Metronidazole-treated brain sections in the 2600–3700 cm^–1^ wave number region normal brain liver sections (Metronidazole treated brain sections spectra-normal brain sections spectra).

It is worth to mention that in the spectral region related to lipids, phospholipids, cholesterol of brain fetus in control and Metronidazole treated samples, no significant differences have been found for some identical peaks, as are shown in Figures 13 and 14. In general for the brain tissues, whilst absorption bands at 699 cm^-1^ (Out-of-plane bending vibrations), 1444 cm^-1^ (δ(CH2), lipids, fatty acids), 1506 cm^-1^ ( amide II), 2911 cm^-1^ (Stretching vibrations of CH2 and CH3 of phospholipids, cholesterol and creatine), 2849 cm^-1^ and 2935 cm^-1^ (C-H stretching bands) were reduced (Figure 15) in Metronidazole treated fetus, the absorption of bands at 843 cm^-1^, 924 cm^-1^ (Left-handed helix DNA (Z form)), 1068 cm^-1^ (stretching C-O ribose), 1122 cm^-1 ^(ν C-O of carbohydrates), 1157 cm^-1^ (C-O stretching vibration), 1556 cm^-1^ (ring base), 1575 cm^-1^ (C=N adenine), 1669 cm^-1^ (amide I) were increased (Figure 16).

## Conclusion

Teratogenesis, as is defined now a day is those new born malformations apparent after birth within a limited period of time. We are yet away from facilities to understand the alterations in fetus biology and structure which are not obvious at the time of birth, or promote later in life like those on hormonal, nervous, liver or kidney functions. Technical and methodological weaknesses are limiting the knowledge of teratogenicity to the very much obvious phenotype malformations right after the birth. Biospectroscopy, as is shown during the last two decades is proving itself of being capable to alert for the biological tissues infra-structural abnormalities in many instances like cancer. Like the developmental history of many other well standardized techniques, FTIR Biospectroscopy is going through its rollercoaster phase; trying to come up with the best method of understanding biological samples spectral meaning and their interpretation. Every new step in understanding its use in different fields is a sign for future vast application of this rather revolutionary technique in medical diagnosis and researchers. This manuscript is one of the first steps toward the use of FTIR biospectroscopy approach on fetus tissues for the diagnosis of teratogenicity. In the present study, the effect of Metronidazole treatment at 60 mg/Kg/day dose in 9th day of pregnancy in mice fetus was investigated at molecular level by using FTIR micro spectroscopy. Liver and brain tissues of fetus exposed with Metronidazole and unexposed liver and brain tissues were determined by comparing with photomicrographs obtained from the haematoxylin and eosin stained tissues. We were successful to trigger spectral peaks of discrimination between a normal and altered fetus by Metronidazole, answering the controversial reports on the teratogenicity of this drug to the level that Metronidazole caused chemical alterations in the biomolecules of fetus liver and brain. As is shown in the result section, Metronidazole has caused many changes in the chemical groups’ composition of mice fetus liver and brain biomolecules. The modifications are very much clear at all important biomolecules of nucleic acids, proteins and fatty acids. The subtractive spectra of spectroscopic regions/related to these three important biomolecules are presented in Figures 8, 9, 15 and 16; which are as the result of mathematical subtraction of the spectra of treated minus normal fetus tissues. As is shown in these figures, not only shifts and breakdown of peaks which might be as the result of structural changes of chemical groups, but the formation of different chemical groups in mice fetus might greatly be affected by Metronidazole. While Metronidazole caused increase in the production of nucleic acid phosphate back bone (around 1200 cm^-1^) and some specific structures in fatty acids (around 2800-2900 cm^-1^), however, has significantly decreased the appearance of some important fatty acids important of the cell membrane.

Yet the present state of Biospectroscopy is limiting a full understanding of the outcome of these biochemical alterations at the present time, but future works might further discover the phenotypic importance of these findings.

This work is clearly indicating the initial tissue infrastructural alterations in two important organs of mice fetus after the exposure of the pregnant mother to Metronidazole which shall be very much important in the early determination of teratogenicity events, and/or discovering those late appearing ignoring teratogenic effects, as described above. Furthermore, FTIR spectroscopy is proposed as a useful and rapid model in the preliminary screening for teratology studies.
